# Central nervous system superficial siderosis: A case report and literature review

**DOI:** 10.1097/MD.0000000000043312

**Published:** 2025-07-11

**Authors:** Qun Xu, Shengnan Wen, Hong Li, Guang Yang, Mingxue Di

**Affiliations:** a Department of Gerontology, The First Affiliated Hospital of Shandong First Medical University & Shandong Provincial Qianfoshan Hospital, Jinan, Shandong, China.

**Keywords:** ataxia, hearing impairment, magnetic resonance imaging, spinal trauma, superficial siderosis of the central nervous system

## Abstract

**Rationale:**

Superficial siderosis of the central nervous system (SSCNS) is a rare condition characterized by iron deposition on the surface tissues of the central nervous system. However, the diagnosis and treatment of diseases lack uniform principles.

**Patient concerns:**

A 64-year-old woman who was admitted to our hospital with progressive difficulty walking.

**Diagnoses:**

Based on the patient’s history of traumatic lumbar fractures, progressive hearing loss, and physical examination findings indicative of cerebellar ataxia, sensorineural deafness, and bilateral pyramidal tract involvement, combined with susceptibility-weighted imaging revealing linear low-signal intensities on the surfaces of the cerebral hemispheres, sulci, and spinal cord, a diagnosis of SSCNS was made.

**Interventions:**

The patient received treatment with Ginkgo biloba leaf extract tablets, mecobalamin, and vitamin B1.

**Outcomes:**

Her symptoms remained stable.

**Lessons:**

A literature review has revealed that most patients with SSCNS exhibit diverse clinical manifestations. Clinicians should consider SSCNS in patients presenting with hearing impairment and gait ataxia, particularly those receiving anticoagulant therapy, brain injury, or surgical intervention. The clinical manifestations, imaging findings, management strategies, and prognosis of these cases are discussed, highlighting the key roles of magnetic resonance imaging and susceptibility-weighted imaging in establishing a definitive diagnosis. In addition, the importance of a multidisciplinary team approach in providing holistic care for individuals has been emphasized. Clinicians are advised to investigate SSCNS in patients with hearing impairment and gait ataxia, particularly in those with a history of brain injury or surgery, using magnetic resonance imaging results.

## 1. Introduction

Superficial siderosis of the central nervous system (SSCNS) is an uncommon neurological condition resulting from chronic, recurrent bleeding into the cerebrospinal fluid (CSF). This leads to the accumulation of iron, primarily as hemosiderin, on the pial surfaces of the brainstem, cerebellum, spinal cord, and cranial nerves.^[1]^ The underlying sources of such chronic hemorrhage are varied and can include central nervous system (CNS) tumors (e.g., ependymomas^[2]^), arteriovenous malformations, dural defects, systemic coagulopathies, and craniospinal traumatic injuries.^[1]^

SSCNS is classically characterized by a triad of progressive sensorineural hearing impairment, cerebellar ataxia, and pyramidal tract dysfunction.^[1]^ However, the simultaneous presentation of all 3 components is infrequent, often complicating and delaying timely diagnosis.^[1]^ Presently, the diagnosis and management of SSCNS face notable challenges, including delayed recognition, a paucity of long-term follow-up data, often inadequate focus on comprehensive etiological assessment, elevated rates of missed diagnoses, and inconsistent treatment standards across different regions.^[1]^ Susceptibility-weighted imaging (SWI) or T2*-weighted gradient-echo magnetic resonance imaging (MRI) sequences are essential for demonstrating the characteristic hemosiderin deposition and establishing the diagnosis of SSCNS.^[1]^

This paper presents a case of SSCNS initially manifesting as cerebellar ataxia and auditory impairment. We examine its etiology, pathogenesis, clinical features, imaging diagnostics, and crucial aspects for fostering early detection. This case underscores the importance of considering SSCNS in the differential diagnosis of patients presenting with progressive cerebellar ataxia or auditory impairment, particularly when accompanied by myelopathic signs, with MRI being pivotal for an accurate diagnosis.

## 2. Case report

This study adhered to the CARE guidelines. The patients’ personal information was anonymized. The patient and their family were notified of the submission of data and images for publication and provided written informed consent. The review board of the First Affiliated Hospital of Shandong First Medical University, Shandong Province, China approved this case for publication.

A 64-year-old female with a university degree was hospitalized because of a year-long progression of walking difficulties, which had intensified over the past 6 months, suggesting a gradual disease process in the CNS. The patient was fully conscious, communicated effectively, exhibited no speech impairment, and could walk independently. She presented with a slightly wide-based gait and impaired mobility alongside a 10-year history of auditory impairment; tinnitus had gradually developed 3 years prior. She sought medical intervention at our facility because her progressively worsening unsteady gait prompted concern. Notably, she suffered a traumatic lumbar fracture in her mid-30s, underwent surgical intervention, and experienced 10 years of worsening auditory impairment along with 1 year of increasing difficulty in ambulation.

The patient was admitted to our department for an additional diagnostic evaluation. Hyperactive tendon reflexes were present in all 4 limbs, resulting from inaccurate bilateral alternating movement, heel-knee-tibia, and finger-nose tests. Romberg’s sign was positive. Maintaining a straight line while walking is difficult. Broad-based gait was also observed. Hoffmann’s sign was positive, whereas Oppenheim’s, Gordon’s, Chaddock’s, and bilateral Babinski signs were negative. In addition, upper-limb postural tremors were observed. Laboratory analyses revealed normal results.

The neurological assessment indicated an minimum mental state examination score of 26. Pure tone audiometry revealed profound sensorineural auditory impairment in the right ear, whereas the left ear exhibited normal hearing. Video head impulse testing yielded positive results for the left horizontal canal test without evidence of otolith involvement. Electromyography revealed damage to the left ulnar nerve at the elbow level and damage to the right median nerve wrist, consistent with mild carpal tunnel syndrome, along with decreased sensory nerve action potential amplitude in the right ulnar nerve. Electromyography ruled out polyneuropathy.

**Figure 1.**
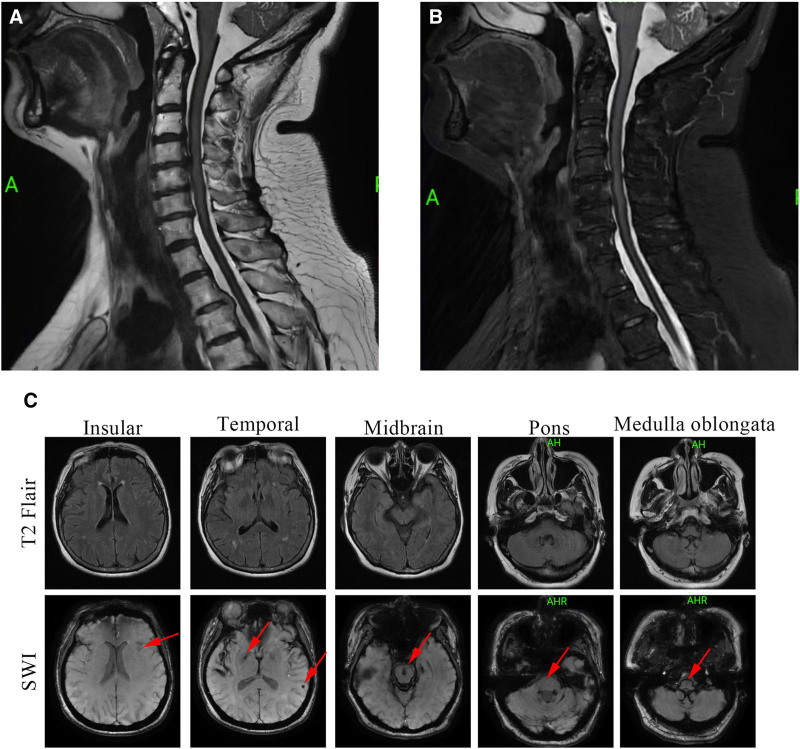
Magnetic resonance imaging shows a toroidal line of low T2 signal on the spinal cord margin on a T2-weighted image, involving the cervical spinal cord (A, B). Brain T2-weighted MRI does not clearly show hemosiderin deposition. Brain MRI using sensitivity weighted imaging (SWI) shows hemosiderin deposits in the bilateral insula, temporal cortex sulci walking area, and around the brain stem (arrows) (C).

**Figure 2.**
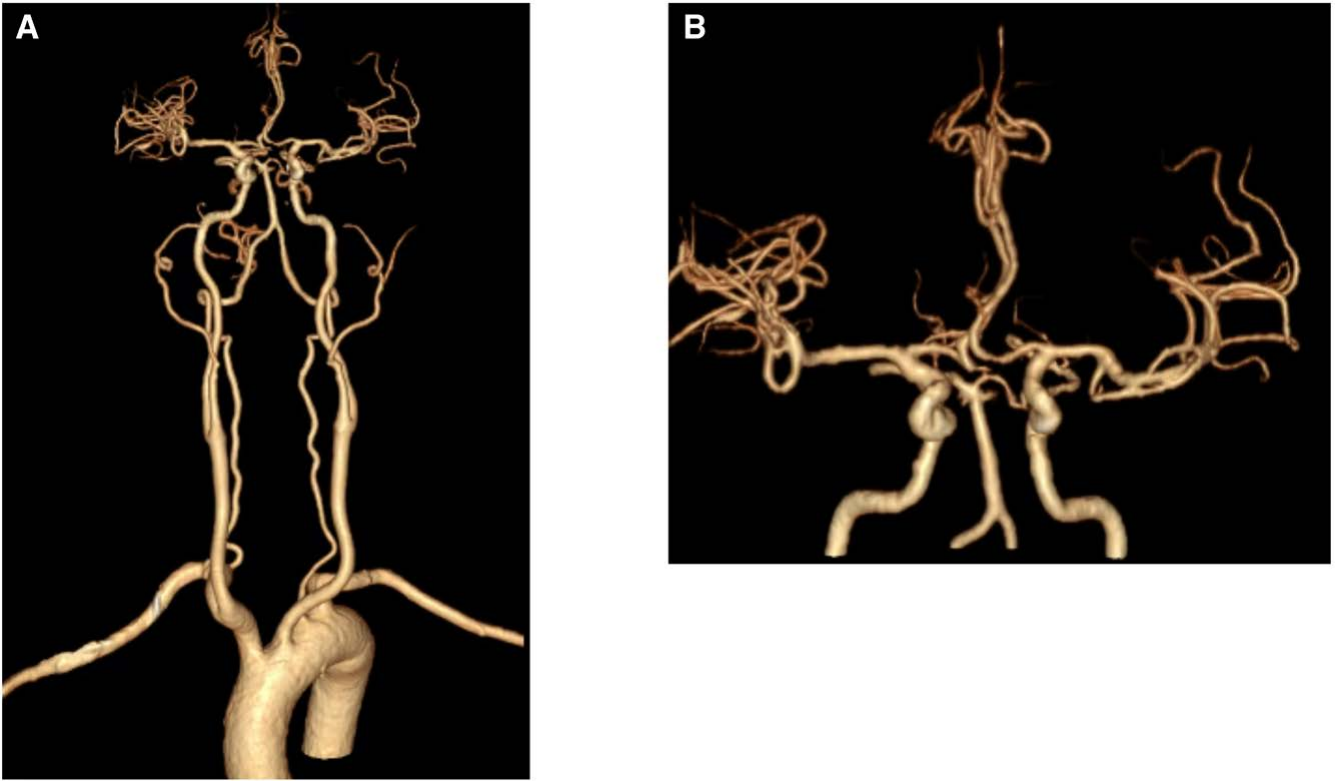
CT angiography performed under craniocervical conditions exhibited no anomalies.

Cervical and craniocerebral MRI showed extensive hemosiderin deposition, highlighting the involvement of the left temporal lobe, brainstem, and cervical pulp. Hemosiderin deposits were visible on standard T2-weighted MRI of the cervical spinal cord (Fig. 1A, B). Hemosiderin deposits are not easily visible on standard T2-weighted MRI, whereas SWI enhances the visibility of these linear hypointense signals across the left temporal lobe, bilateral insula, temporal cortex sulci, and the brainstem (Fig. 1C). CT angiography performed under craniocervical conditions showed no anomalies (Fig. 2A, B).The chest CT on July 17th before the patient’s admission and the current abdominal and pelvic CT showed no abnormalities in the thoracic and lumbar vertebrae (Figure S1, Supplemental Digital Content, https://links.lww.com/MD/P393).

**Figure 3.**
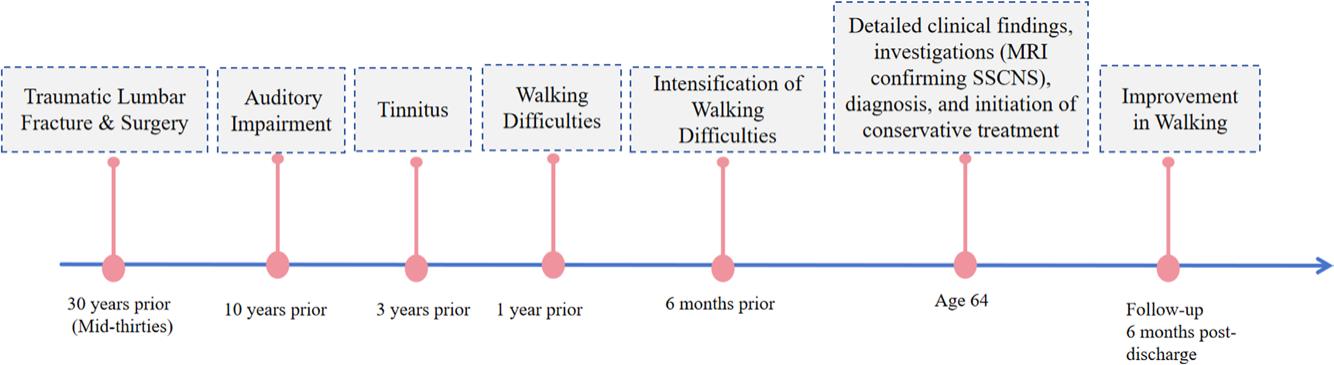
Clinical timeline of the patient.

The patient refused all invasive procedures such as lumbar puncture, CT myelography or lumbar vascular digital subtraction angiography. A multidisciplinary team from neurology, otolaryngology, and rehabilitation therapy conservatively assessed her condition to enhance balance, gait, and upper limb function. Based on the above tests and examinations, we made a differential diagnosis (summarized in Table 1). The final diagnosis was classical SSCNS. With the patient’s family’s consent, a treatment regimen consisting of Ginkgo biloba extract, mecobalamin, and vitamin B1 was initiated. 6 months after discharge, the patient walked better than before the discharge, without adverse events. The patient’s clinical timeline was showed in Figure 3,which outlines key events and interventions in the patient’s care.

**Table 1:** Differential diagnosis and exclusion criteria.

Alternative diagnosis	Reason for exclusion
Vestibular disorders (e.g., Vestibular Schwannoma)	Pure tone audiometry confirmed profound sensorineural hearing loss (right ear). VHIT positive for left horizontal canal, but MRI did not show evidence of a vestibular schwannoma or other structural inner ear/vestibular nerve pathology that would typically explain such profound unilateral deafness and progressive ataxia. The ataxia was also more cerebellar in nature (wide-based, impaired heel-knee-tibia, etc) than purely vestibular.
Common cerebellar ataxias (e.g., Spinocerebellar Ataxias, Multiple System Atrophy)	While progressive ataxia was present, the MRI findings of extensive, characteristic hemosiderin deposition on SWI/T2* along brain and spinal cord surfaces are pathognomonic for SSCNS and not typical for others. The specific combination with profound unilateral sensorineural hearing loss is also more suggestive of SSCNS.
Brainstem lesions (e.g., Stroke, Tumor, Demyelinating Disease)	While the brainstem was involved (hemosiderin deposition), craniocervical CTA showed no vascular anomalies (ruling out acute/subacute stroke as the primary progressive cause). MRI did not show features typical of primary brainstem tumors or demyelinating plaques. Instead, the specific pattern of superficial hemosiderin deposition pointed to SSCNS.
Cervical myelopathy (due to other causes e.g., Spondylosis, Tumor, Syringomyelia)	Although signs of myelopathy were present (active reflexes, positive Hoffmann’s), the cervical MRI showed superficial hemosiderin deposition along the cervical cord, consistent with SSCNS, rather than intrinsic cord compression from severe spondylosis, an intramedullary tumor, or syringomyelia as the primary pathology.
polyneuropathy/peripheral neuropathy	Electromyography ruled out polyneuropathy. While it revealed focal neuropathies (left ulnar, right median – carpal tunnel), these findings do not explain the profound cerebellar ataxia, sensorineural hearing loss, or the widespread CNS hemosiderin deposition.
Vitamin deficiencies (e.g., B12)	Laboratory analyses were reported as normal, presumably including relevant vitamin levels if suspected clinically. The MRI findings are not typical for B12 deficiency myelopathy.

CNS = central nervous system, MRI = magnetic resonance imaging, SSCNS = superficial siderosis of the central nervous system, SWI = susceptibility-weighted imaging, V-HIT = Video head impulse testing.

## 3. Discussion

A literature review was performed using a database search for studies published up to November 1, 2024, using the search terms “superficial siderosis” and “central nervous system.”

SSCNS is an uncommon disorder characterized by subarachnoid iron deposition, usually caused by persistent or intermittent chronic bleeding from small blood vessels or capillaries.^[3]^ SSCNS is a relatively rare but underdiagnosed clinical syndrome, characterized by progressive, symmetrical, bilateral sensorineural auditory impairment, ataxia, myelopathy, and cognitive dysfunction.

Wilson categorized SSCNS into cortical shallow siderosis (SS) and infratentorial shallow siderosis (ISS), based on the location of hemosiderin deposition. Cortical superficial siderosis primarily accumulates on the cerebral cortex surface and is often linked to neurological disorders such as Alzheimer’s disease. Common causes include reversible cerebral vasoconstriction syndrome, vasculitis, high-grade proximal artery stenosis, thrombosis of cerebral veins or sinuses, aneurysm-related subarachnoid hemorrhage, amyloid angiopathy, and other forms of recurrent or isolated subarachnoid hemorrhage.^[4]^ ISS is characterized by bilateral siderosis affecting the surface of at least 2 regions, including the brainstem (midbrain, pons, and medulla oblongata), cerebellum (cerebellar lobes, peduncles, and vermis), spinal cord, and craniocervical junction, and may extend supratentorially.^[5]^ Key features include sensorineural auditory impairment, cerebellar ataxia, and pyramidal signs.^[1]^ This discussion focuses solely on ISS.

### 3.1. Pathogenesis

The initial epidemiological understanding of SSCNS is largely based on a comprehensive literature review conducted in 1995. Chronic subarachnoid hemorrhage is the predominant cause of disease,^[1]^ such as prior hemorrhage, trauma, surgery, tumors, or vascular abnormalities.^[3]^ Ventral dural defects may have various etiologies.^[3]^ Neuronal damage in SS is caused by unbound iron, which occurs when the biosynthetic capacity of microglia for ferritin and hemosiderin is exceeded.^[6]^ Hemosiderin has accumulated on the brain’s surface, brain stem, cerebellum, cranial nerves VIII, and spinal cord for decades, causing free iron toxicity in the surrounding tissues,^[4]^ which triggers the release of HO-1 and ferritin from neurons. HO-1 breaks down free hemoglobin into iron, biliverdin, and carbon monoxide. The resulting ferritin and free iron form hemosiderin, which mediates oxidative stress and free radical production, ultimately causing neuronal death and compromising the structural and functional integrity of nerve tissue.^[7]^ In patients with SS, the latency period between injury and symptom onset ranges from 8 to 37 years.^^[3]^^

### 3.2. Clinical manifestations

CNS siderosis typically presents as a constellation of neurological symptoms that progressively worsen over time. The primary symptoms of ISS are auditory impairment and ataxia, whereas in cortical SS, cognitive impairment and seizures are predominant symptoms.^[5]^ Clinical symptoms generally correlate with the anatomical distribution, progression, and individual variability of siderin deposits in the CNS, affecting areas such as the vermis, superficial sulci, gyri, basal frontal lobe, temporal lobe, brainstem, spinal cord, and cranial nerves I, II, and VII. The extended glial segment of the vestibulocochlear nerve makes it particularly susceptible to axonal injury due to iron deposition.^[8]^ These symptoms culminate in a characteristic combination of irreversible sensorineural auditory impairment, progressive cerebellar ataxia, and pyramidal signs.^[3]^ Auditory impairment, often preceded by tinnitus, is the earliest and most prevalent symptom of SSCNS, and typically progresses to sensorineural auditory impairment and deafness within 15 years. Age-inappropriate auditory impairment was also observed in this case. Ataxia, a loss of voluntary muscle coordination, often presents as unsteady gait or a general lack of motor coordination and is frequently the first symptom prompting patients to seek medical attention. It is often an indicator of severe progressive neurological decline caused by the destructive effects of iron deposition in cerebellar tissue, particularly affecting the Purkinje cells and cerebellar lobes. Therefore, early recognition of ataxia is essential for timely diagnosis of SSCN.

Other findings included cognitive impairment (50%), amnesia, headache, seizures, myoclonus, visual disturbances, hypopnea (17%), paresthesia (13%), dysphagia, bladder and bowel disorders (24%), symptoms of intracranial hypotension,^[3]^ hyperactive dyskinesia^[9]^ and kinetic tremor.^[4]^ Reports of neck or back pain, bilateral sciatica, and lower motor neuron signs are rare.

Peripheral vestibular dysfunction aggravates patient imbalance.^[10]^ Electronystagmography and video head impulse testing examinations revealed no vestibular function impairment in the patient.

### 3.3. Differential diagnosis and diagnosis

Differential diagnosis encompasses a broad spectrum of conditions such as progressive auditory impairment, cognitive decline, ataxia, and distinctions between pyramidal and extrapyramidal signs, including, but not limited to, chronic subarachnoid hemorrhage, neurodegenerative disorders, and malignancies affecting the brain or spinal cord.

Tremor in SSCNS predominantly manifests as an action tremor, often exhibiting features of intention tremor (cerebellar tremor), and is frequently accompanied by ataxia. This tremor is typically characterized by a low frequency (e.g., 3–5 Hz) and high amplitude, and may coexist with other neurological deficits, such as myoclonus or pyramidal tract signs. In contrast, essential tremor classically presents as a postural and kinetic tremor, with a higher frequency (e.g., 4–12 Hz) and lower amplitude.^[11]^ Given that essential tremor is a diagnosis of exclusion, secondary tremor etiologies, including SSCNS, require diligent investigation and exclusion.

CSF markers of subarachnoid hemorrhage (e.g., hemosiderin-laden macrophages) support the etiology of siderosis, but definitive SSCNS diagnosis requires neuroimaging, especially SWI MRI, to demonstrate characteristic superficial iron deposition on the brain and spinal cord. Early diagnosis is crucial, and efforts should focus on locating the bleeding source since treatment may be possible. CSF biomarkers can aid in the diagnosis and monitoring of iron deposition disorders in the CNS. Elevated levels of tau protein, lysosomal acid lipase activity, β-amyloid, and glial fibrillary acidic protein, along with increased red blood cells and high iron and ferritin concentrations, may serve as disease biomarkers, reflecting the body’s response to iron accumulation.^[12]^ However, in most cases, CSF appears normal.^[12]^ Since its initial characterization using MRI in 1985, MRI has become the diagnostic gold standard for SS because of the high sensitivity of T2-weighted gradient recall echo and SWI sequences for hemosiderin deposition.

Surgical intervention can fundamentally treat vascular diseases, including malformations, by addressing underlying causes and halting disease progression. digital subtraction angiography remains the gold standard for cerebrovascular disease screening and provides greater accuracy than magnetic resonance or computed tomography angiography.

The patient presented with chronic progressive ataxia and worsening sensorineural auditory impairments. MRI confirmed the pathology, showing iron deposition on the surfaces of the brain, brainstem, and spinal cord, along with signal loss on T2-weighted images. Imaging findings, combined with chronic progression of the patient’s symptoms, raised the suspicion of SSCNS. However, the exact source of hemorrhage in this case remains undetermined. The patient declined further invasive operation to confirm cerebrovascular hemorrhage or malformation, and a treatment plan was formulated based on this condition.

### 3.4. Treatment

Surgical intervention may be employed to remove or repair chronic subarachnoid hemorrhage caused by cerebrovascular malformations, tumors, or other causes.^[13]^ If the dural defect is repaired via a posterior surgical approach, leakage into the subarachnoid space can be effectively prevented, potentially halting its progression.^[14]^

In addition to surgical intervention, medical therapy often includes supportive care to address symptoms, such as cerebellar ataxia and neuropsychological deficits. Additionally, removal of iron deposits from the brain may improve neurological function and support recovery. Studies have reported the use of fat-soluble iron chelators such as deferiprone, deferoxamine, and triethylenetetramine (trientine) to stabilize disease progression. In Kessler’s 2-year prospective study, MRI indicated a significant reduction in hemosiderin deposition, with some symptom improvement.^[15]^ However, some studies have found that deferiprone’s benefits may not outweigh its risks, with agranulocytosis being the most concerning complication associated with its use.^[16]^ Symptomatic treatments include hearing aids for deafness, and physical therapy for ataxia to improve patient functionality and quality of life.

It is necessary to study the treatment of iron deposition in the CNS with traditional Chinese medicine (TCM). According to the pathological mechanism, the inhibition of cell ferroptosis and oxidative stress may slow down the progression of SSCNS. A variety of TCMs play the roles of reducing oxidative stress, promoting nerve repair and improving iron metabolism in tumors, diabetes and nerve injury.^[17–22]^ Current research has found that the pathogenic mechanism of SSCNS may involve multiple aspects mentioned above. These research results provide an important theoretical basis for the study of TCM in the treatment of SSCNS.

Ginkgo biloba extract is widely used for neuroprotection and circulation. It is believed to have antioxidant properties, improve microcirculation (including blood flow in the brain and vestibule), and regulate the neurotransmitter system. It has been studied for cognitive impairment, dementia, vertigo, tinnitus and peripheral artery disease.^[21,23]^ The persistent iron-mediated oxidative stress in the pathological mechanism of SSCNS can stimulate nerve damage, while ginkgo biloba extract alleviates the above process by inhibiting oxidative stress. Meanwhile, ginkgo biloba extract may improve cerebellar function or alleviate ataxia and relieve some symptoms of SSCNS such as tinnitus by enhancing cerebral and cerebellar blood flow or neuroprotective effects.

TCM usually aims to restore balance and support healing. Practitioners may recommend herbal or acupuncture treatments to alleviate the symptoms of chronic neurological diseases. If the patient temporarily refuses invasive treatment and adopts TCM treatment, the secondary symptoms caused by SSCNS can be alleviated and regulated to improve the patient’s quality of life, rather than directly removing the deposited hemosiderin. However, the application of TCM to SS requires caution. It should be regarded as an adjunctive treatment to conventional evidence-based treatment and be supervised by qualified TCM practitioners in consultation with the primary neurology team. Further rigorous research is necessary to determine the safety and efficacy of TCM for SSCNS.

Mecobalamin is the active coenzyme form of vitamin B12. It plays a crucial role in nucleic acid synthesis, myelin formation and neuronal function. It is usually used to treat peripheral neuropathy (diabetes, alcohol, drug-induced), vitamin B12 deficiency, and sometimes as an adjunctive treatment for other neurological diseases.^[24]^ The electromyogram of this patient showed focal peripheral nerve injury (left ulnar nerve, right median nerve). Mecobalamin can provide treatment for these peripheral nerve problems. Considering spinal cord symptoms (pyramidal tract involvement), mecobalamin is used as a general neural support application. The application in this case aims to optimize peripheral nerve health and provide general metabolic support for the nervous system.

Vitamin B12 (thiamin) is an essential coenzyme in carbohydrate metabolism and is crucial for normal neuronal function, including nerve impulse conduction and the maintenance of myelin sheath.^[25]^ Because the patient presented with obvious cerebellar ataxia. Although not due to thiamin deficiency, ensuring adequate thiamin is important for optimal cerebellar and overall neurological function.

Despite treatment, some patients continue to experience SS progression, potentially due to the irreversible neurodegenerative cascade initiated by prolonged iron deposition.

### 3.5. Prognosis and follow-up

SSCNS typically progresses with a gradual decline in neurological function over years or decades, often leading to poor outcomes in the absence of treatment. Early identification and management of potential bleeding sources, combined with prompt diagnosis and treatment, are essential for improving patient prognosis.

The diagnosis and management of SSCNS requires a multidisciplinary approach, with essential contributions from neurology, radiology, rehabilitation medicine, and other specialties. Regular clinical evaluations are crucial for monitoring the progression of symptoms such as auditory impairment, ataxia, and myelopathy.

### 3.6. Limitations

The follow-up period for the patients with SSCNS was limited. Diverse initial symptoms can be mild, making early medical consultation challenging for some patients. Based on the literature and the diagnosis and treatment process in this case, SSCNS should be strongly suspected in the following clinical scenarios: (1) history of hemorrhage, trauma, tumor, or CNS inflammation; (2) unexplained cerebellar ataxia with progressive sensorineural auditory impairment; and (3) unexplained cognitive impairment, particularly rapidly progressing dementia. In cases without a clear history of cerebral hemorrhage or trauma, etiological evaluations such as CSF analysis and cerebral angiography are recommended. Prompt treatment following diagnosis is essential for an effective intervention.

## Author contributions

**Conceptualization:** Mingxue Di.

**Investigation:** Qun Xu.

**Project administration:** Guang Yang, Hong Li.

**Resources:** Guang Yang, Hong Li.

**Writing – original draft:** Qun Xu, Shengnan Wen, Guang Yang, Hong Li.

**Writing – review & editing:** Mingxue Di.

## Supplementary Material

SUPPLEMENTARY MATERIAL
