# Supplementary material for: Central nervous system superficial siderosis: A case report and literature review
Source: Medicine (Baltimore). 2025 Jul 11;104(28):e43312. doi: 10.1097/MD.0000000000043312 (PMC12262984; doi:10.1097/MD.0000000000043312)

Supplement Figure 1：Chest CT and abdominal & pelvic CT showed no abnormalities in the thoracic and lumbar vertebrae.


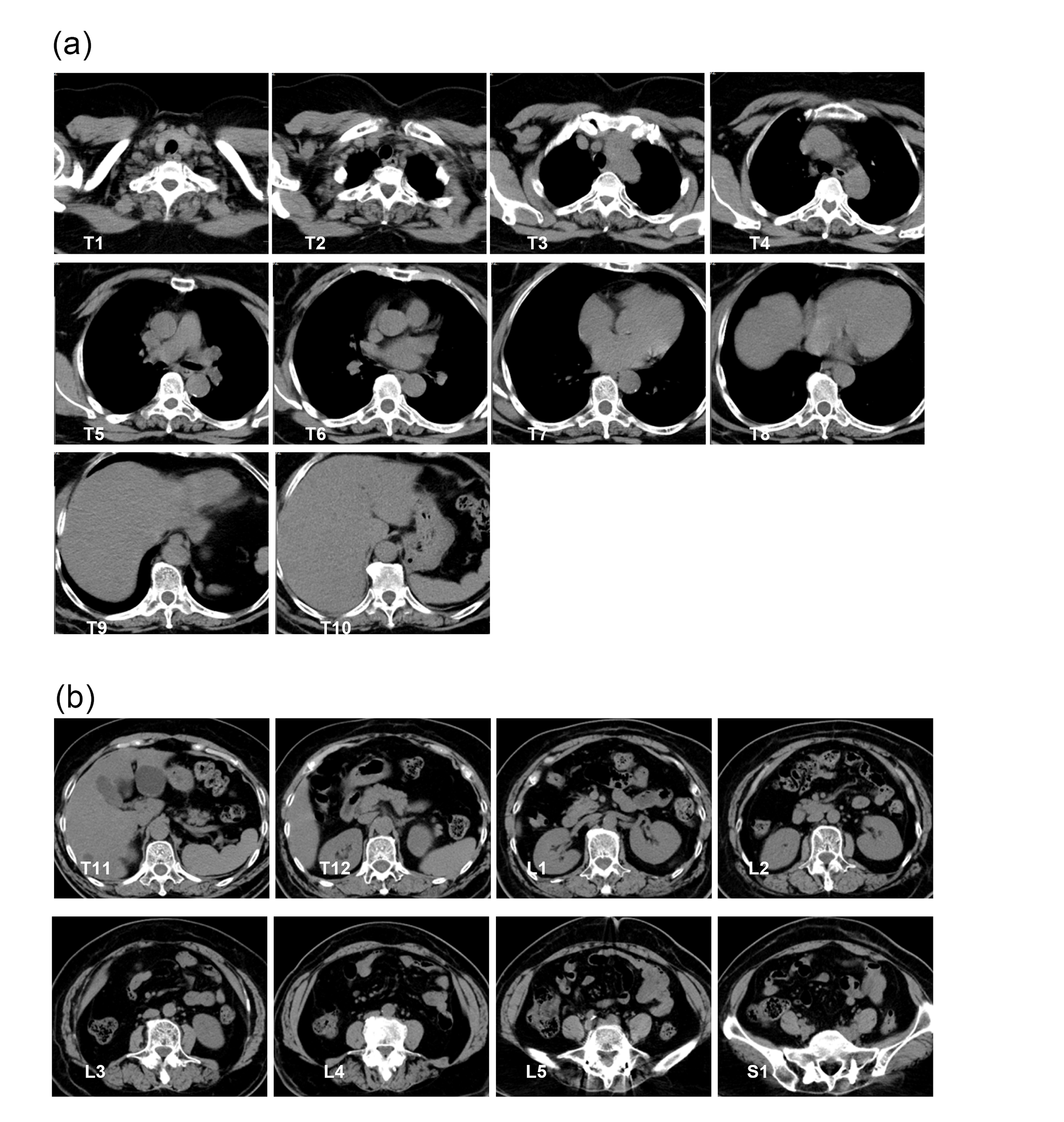

Supplement: SUPPLEMENTARY MATERIAL [file medi-104-e43312-s001.docx]
